# The Effect of Personal Characteristics, Perceived Threat, Efficacy and Breast Cancer Anxiety on Breast Cancer Screening Activation

**DOI:** 10.3390/healthcare5040065

**Published:** 2017-09-27

**Authors:** Patrick De Pelsmacker, Martine Lewi, Veroline Cauberghe

**Affiliations:** 1 Department of Marketing, Faculty of Applied Economics, University of Antwerp, Prinsstraat 13, 2000 Antwerpen, Belgium; tine.lewi@gmail.com; 2 Department of Communication Sciences, Ghent University, Korte Meer 7-11, 9000 Gent, Belgium; veroline.cauberghe@ugent.be

**Keywords:** breast cancer screening activation, personal characteristics, perceived threat, perceived efficacy, evoked anxiety

## Abstract

In order to activate women to participate in breast cancer screening programs, a good understanding is needed of the personal characteristics that influence how women can be activated to search for more information, consult friends and doctors, and participate in breast cancer screening programs. In the current study, we investigate the effect of six personal characteristics that have in previous research been identified as important triggers of health behavior on breast cancer screening activation: Health awareness, Need for Cognition, Affect Intensity, Breast cancer knowledge, Topic involvement, and the Perceived breast cancer risk. We test the effect of these factors on four activation variables: intention of future information seeking, forwarding the message to a friend, talking to a doctor, and actual breast cancer screening attendance. Additionally, we try to unravel the process by means of which the antecedents (the six personal characteristics) lead to activation. To that end, we test the mediating role of perceived breast cancer threat, perceived efficacy of screening, and the evoked breast cancer anxiety as mediators in this process. The data were collected by means of a cross-sectional survey in a sample of 700 Flemish (Belgium) women who were invited to the free-of-charge breast cancer population screening. Screening attendance of this sample was provided by the government agency in charge of the organisation of the screening. Health awareness, affects intensity, topic involvement, and perceived risk have the strongest influence on activation. Breast cancer anxiety and perceived breast cancer threat have a substantial mediation effect on these effects. Efficacy perceptions are less important in the activation process. Increased health awareness and a higher level of perceived risk lead to less participation in the free of charge population based breast screening program. Implications for theory and practice are offered. The limitation of the study is that only a standard invitation message was used. In future research, other types of awareness and activation messages should be tested. Additionally, the analysis could be refined by investigating the potentially different activation process in different subgroups of women.

## 1. Introduction

One in seven women are confronted with breast cancer over their lifespan [1]. Early diagnosis can lead to less severe consequences. This has been one of the main drivers for establishing the population based breast cancer screening in most developed countries. The free of charge population-based breast cancer screening program among women aged 50 to 69 has been offered in Flanders (Belgium) since June 2001. The average participation rate has been increasing over the past few years, but the target participation rate of 75% has not been reached yet. In order to further increase the participation rate, a good understanding is needed of the drivers of breast cancer screening activation amongst the target group. 

One specific strategy to motivate cancer prevention and early detection behaviors is to tailor health messages to the characteristics of the target group. Latimer et al. [2] and Williams-Piehota et al. [3] studied message tailoring in the context of mammography screening participation and found that messages that were tailored to individual variability are more persuasive in promoting screening mammography than mismatched messages. They conclude that there is evidence for the usefulness of “psychological tailoring” as a health communication strategy and a direction for developing effective health messages. Psychological characteristics of individuals are essential criteria in the design of the screening appeals [4]. However, little research has been conducted on how personal characteristics have an effect in a health prevention context. 

Prior research has identified a number of personal characteristics of the target group that can have an impact on health behavior and decisions [2,3]. Three intrinsic individual characteristics have emerged as important antecedents of these behaviors and decisions: health awareness, need for cognition, and affect intensity. For instance, several authors found a positive relationship between health awareness and preventive healthcare behaviors [5,6,7]. Prior research has found a relationship between the need for cognition and information seeking and subsequent information processing: individuals high in the need for cognition expand more cognitive efforts on information search [8,9], also in the context of breast cancer screening [3,10]. It has been observed that differences in affect intensity are related to differences in affective, cognitive, and behavioral responses both in a general consumer and health context [11,12,13,14,15], showing that affect intensity has an effect on the activation of individuals [16,17]. Additionally, three breast cancer specific personal characteristics have been identified as particularly relevant: breast cancer knowledge, breast cancer topic involvement, and perceived breast cancer risk. For instance, previous research has revealed a relationship between health literacy and health attitude, specifically regarding cancer [18]. Other studies have found a significant relation between topic involvement and adherence to screening recommendations [19]. Finally, perceived risk has been extensively investigated in prior health communication research and specifically in the area of breast cancer [20,21,22,23,24]. The first aim of the current study is to investigate the effect of these six personal characteristics on breast cancer screening activation.

Most previous studies have focused on behavioral outcomes such as actual screening attendance. However, the actual attendance is only one aspect of activation and it may be the end result of other, intermediary, forms of activation, such as being triggered to look up more information, forward a message to friends, or visit a doctor. These types of activation may be important because they may eventually lead to more awareness and actual screening behavior. An important theoretical contribution of our work is that it explicitly takes these other activation behaviors (or intentions thereto) into account, something that has not received much attention in previous studies.

Another important knowledge gap we try to fill, and for which there is only scant previous research, is to unravel the mechanism behind the effects of personal characteristics on breast cancer screening activation. Testing the mental processes through which personal characteristics have an activation effect, is an essential contribution to insight into the mechanism of activation. Based on the protection motivation and the extended parallel processing model, we explore to what extent evoked threat, anxiety, and perceived efficacy mediate the effect of personal characteristics on activation. 

**Figure 1:**
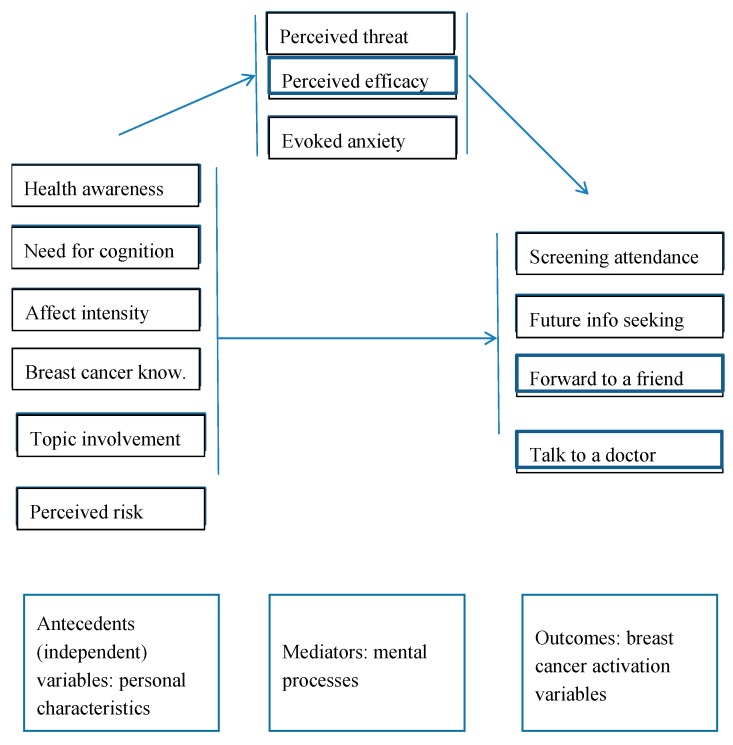
Conceptual framework.

In summary, in the current study, we investigate the effect of six intrinsic and topic-specific personal characteristics on breast cancer screening participation activation: health awareness, need for cognition, affect intensity, prior breast cancer knowledge, topic involvement, and perceived risk of breast cancer. A mediation model is proposed and tested of the effects of personal characteristics on perceived threat and efficacy and breast cancer anxiety (mediators), and to what extent the latter processing variables have an effect on breast cancer screening activation. The conceptual framework of the study is shown in Figure 1. The study was carried out in Belgium, in a sample of 700 women aged 50–69 who were invited to participate in free-of-charge breast cancer screening, and completed a questionnaire accompanying this invitation. The theoretical contribution of the current study is that it investigates the effect of different personal characteristics on a number of previously not yet studied behavioral activation variables in the area of breast cancer screening. Further, it documents the mental processes by means of which perceived threat and efficacy and the evoked anxiety lead to activation in women differing in personal characteristics. The study also informs policy makers to fine-tune screening awareness and activation campaigns. 

## 2. Literature Review, Hypotheses and Research Question

Six personal characteristics are studied in the current study, based on their potential relevance for breast cancer screening attendance as well as on other activation variables: intention to seek breast cancer information in the future, forward the message to a friend, talk to a doctor, and to participate in the population based breast cancer screening. In the hypotheses and research question, we refer to ‘breast cancer screening activation’ to indicate these four dependent variables.

Health awareness or health consciousness refers to the degree to which health concerns are integrated into a person’s daily activities [5]. Jayanti and Burns [5] found a positive relationship between health consciousness and preventive healthcare behaviors. Gould et al. [6] also found that health awareness is one of the predicting factors for a more positive approach to healthcare and prevention. Chang [7] observed a difference in message processing among individuals who are concerned about a health threat, as compared to the individuals who are not. In the context of hepatitis B awareness campaigns, concern increased the level of perceived message effectiveness. This was not the case for individuals who were not concerned about hepatitis B. As previous research indicates that a higher level of health awareness increases message acceptance, the following hypothesis is formulated.

### 2.1. Hypothesis 1. A higher Degree of Health Awareness Leads to a Stronger Breast Cancer Screening Activation than a Lower Degree of Health Awareness

*Need for cognition* (NFC) is the intrinsic desire or the individual tendency to engage in challenging intellectual activity and enjoy thinking [9,25]. Prior research has found a relationship between NFC and attitudes towards information, such as information seeking and subsequent information processing. High NFC-individuals select more information and generate more task-related cognitive responses when compared to low NFC-individuals. Moreover, high NFC-individuals expand more cognitive effort on information search than low NFC-individuals [8,26]. The anticipation of engaging in intellectual activity seems to stimulate different motives in people with a high and low need for cognition and the mindset induced by these motives seems to influence behavioral decisions [9]. Prior research shows that message appeals only result in adaptive coping among respondents who are in high need for cognition [19]. In the context of mammography screening and the impact of tailoring persuasive health communications about mammography screening, Williams-Piehota et al. [3,10] observe that messages matched to an individual’s NFC are more influential than mismatched messages. Specifically, matched messages are better at motivating mammography among high-NFC women. Therefore, it is expected that NFC has a positive effect on breast cancer screening activation behavior, leading to following hypothesis:

### 2.2. Hypothesis 2. A Higher Degree of NFC Leads to a Stronger Breast Cancer Screening Activation, than a Lower Degree of NFC

*Affect intensity* (AI) is a stable individual difference in the strength with which individuals experience their emotions [11]. Affect intensity is positively related to affective, cognitive, and behavioral responses in various contexts [11,27]. Substantial research has been carried out into the differences in processing information, more specifically the emotional aspects, by people with either high or low affect intensity [28], both in a general consumer and a health context [13,14,15]. Although there is limited prior research in the domain of health communication, it can be expected, based on prior research [16,17], that higher affect intensity has a positive effect on the activation variables. Therefore, the following hypothesis is formulated: 

### 2.3. Hypothesis 3. A Higher Degree of Affect Intensity Leads to a Stronger Breast Cancer Screening Activation than a Lower Degree of Affect Intensity

*Breast cancer knowledge* is the prior knowledge the individual has gathered and makes up the current understanding about broad aspects of breast cancer, such as screening, symptoms, treatments, etc. There is an association between low health literacy (and, more, specifically, low cancer knowledge) and variables that affect the engagement in cancer prevention and screening activities. Prior research shows that adults with low health knowledge demonstrate a less proactive health attitude, specifically regarding cancer. This is expressed in avoiding doctor’s visits more often and having more fatalistic attitudes toward cancer. Low health literacy also leads to being less accurate in identifying the purpose of cancer screening tests and being more likely to avoid information about diseases a person did not encounter yet [18] Given the above prior research on knowledge and literacy, the following hypothesis is advanced: 

### 2.4. Hypothesis 4. A Higher Degree of Prior Breast Cancer Knowledge Leads to a stronger Breast Cancer Screening Activation than a Lower Degree of Prior Knowledge

*Topic involvement* is the degree to which an individual is involved with the topic, in this case, breast cancer. Individuals can have a higher involvement due to prior experience with the disease within the individual’s community or a higher actual risk. Involvement can also be established and further encouraged, e.g., through memorable messages. Smith et al. [29] investigated whether memorable messages can promote protection against breast cancer and guide health behaviors. Participants in the study were asked to report their personal, friends’, and relatives’ experiences with breast cancer and a memorable message about breast cancer if one came to mind. Individuals who had personal and friend or relative experience with breast cancer were significantly more likely to recall memorable messages than other respondents, and thus were more likely to take these messages into account. Rutten and Iannoti [19] investigated beliefs and characteristics among women with and without a breast cancer family history and involvement with breast cancer issues. They especially study the topic of adherence to annual mammography screening recommendations. They report a significantly positive relation between issue involvement and adherence. Findings by Petty and Cacioppo et al. [26] also indicate that, under high involvement, individuals tend to be more willing to elaborate about the message. Therefore, a higher topic involvement is expected to lead to more breast cancer screening awareness activation and therefore following the hypothesis is advanced: 

### 2.5. Hypothesis 5. A Higher Degree of Topic Involvement Leads to a Stronger Breast Cancer Screening Activation than a Lower Degree of Topic Involvement

*Perceived risk* is the perception of an individual about his/her personal risk or chance to develop a disease, for instance due to a breast cancer family history or prior breast cancer experience [20]. Women with a personal or maternal history of breast cancer can be vulnerable to higher levels of psychological stress in relation to breast cancer risk. Berlin et al. and Nan et al. [20,30] investigated the effects of perceived risk on the seeking of breast and prostate cancer information. Greater perceived risk was predictive of more information seeking. Lipkus et al. [23] investigated the extent to which informing women about their risk for breast cancer affects their perceived 10-year and lifetime risks for getting breast cancer, their emotional reactions toward getting breast cancer, and their intentions to get mammograms. The results indicate that, overall, women reported that upon obtaining their 10-year risk estimate, either did not affect or increase their intentions to get mammograms. The authors conclude that giving women their individual risk of getting breast cancer enhances their feelings that they are at lower risk than other women. Reducing women’s perceived risk of breast cancer did not lower their intentions to get mammograms. Bolton et al. [31] states that a decline of breast cancer screening participation could be largely attributable to reductions in screening visits by women who are at a low risk of developing breast cancer. Walker et al. [32] conclude that a small positive association has been consistently demonstrated between perceived breast cancer risk and mammography use. In their meta-analysis examining the association between perceived breast cancer risk and an adherence to mammography, clinical breast examination or breast self-examination guidelines among women with familial breast cancer risk, they report a weak positive association between a higher perceived risk and an adherence to mammography guidelines. No consistent association was found between perceived risk and the adherence to clinical breast examination or breast self-examination guidelines. A meta-analysis by Katapodi et al. [24] examined, amongst others, the relationship between perceived risk and breast cancer screening. The results show that women do not have accurate perceptions of their breast cancer risk. In general, women have an optimistic bias about their personal risk. They conclude that there is an association between perceived risk and mammography screening. This study was one of the first to demonstrate that women who perceive a higher breast cancer risk are more likely to pursue genetic testing or undergo prophylactic mastectomy. However, it is unclear whether perceived risk also influences adherence to breast self-examination. However, Bowen et al. [22] found no relationship between perceived breast cancer risk and mammography use. Gallagher et al. [33] examined how the beliefs about risk shape responses to messages about cancer screening. The authors did not find that women’s perception of susceptibility to developing breast cancer was associated with either their construal of the function of mammography or their perceived risks associated with screening. However, it should be noted that the majority of the women in the study construed mammography as an illness-detecting behavior, emphasizing that even women who perceived a lower susceptibility to breast cancer were likely to construe a mammogram as a test that serves to detect a problem rather than affirm that they are healthy. 

Some previous research also points at the negative effects of risk perception on breast cancer screening activation. Kash et al. [34] investigated the beliefs of women at high risk for breast cancer (one or more first-degree relatives with breast cancer) about their breast cancer risk and the impact of this information on their surveillance behaviors. High risk perception predicted a poor adherence to monthly breast self-examination. More than 27% of the women at high risk were defined as having a level of psychological distress, according to the authors, “consistent with the need for counseling”. Women reporting more barriers to screening, fewer social support, and low social desirability had more psychological distress. Higher anxiety was directly related to poor attendance at a clinical breast examination and poor adherence to monthly breast self-examination. These results suggest that a high risk perception may lead to less activation, and that this effect is mediated by developing high levels of anxiety. 

Although there is some lack of consistency in the results of previous research regarding the relationship between perceived breast cancer risk and awareness activation and screening attendance, the majority of the findings in previous studies point at a positive relationship. The following hypothesis is advanced: 

### 2.6. Hypothesis 6. A Higher Degree of Perceived Risk Leads to a Stronger Breast Cancer Screening Activation than a Lower Degree of Perceived Risk

In the previous sections, we have developed hypotheses with respect to the relationship between personal characteristics and breast cancer screening activation. An interesting question is which mental processes explain these presumed effects. Different theories have been proposed on how intrinsically threatening messages (such as the one in this study) influence the activation of the receiver of the message, and through which processes. The *fear-as-acquired drive model* [35] claims that some fear arousal is needed to elicit a motivational drive state (i.e., create tension), but too much fear would result in maladaptive outcomes (e.g., defensive avoidance). Hence, a moderate amount of fear arousal would produce the most positive attitude change. However, consecutive tests of this fear theory led to its rejection [36]. The *Protection Motivation Theory (PMT)* [37] defines four cognitive reactions as response to a fear appeal: perceived severity of and perceived susceptibility to the threat (together: perceived threat), response efficacy, and self-efficacy (together: perceived efficacy). These cognitive responses lead to “protection motivation”. Increases in threat severity, threat vulnerability, response efficacy, or self-efficacy facilitate adaptive intentions or behaviors [38]. The PMT has been applied extensively to various domains of health communication, such as adherence to medical-treatment regimens [38], and smoking cessation [39]. In their meta-analysis, Milne and Orbell [40] confirm that, overall, the components of PMT are predictive of health-related intentions. The *Extended Parallel Process Model (EPPM)* integrates the fear drive model and the PMT model [36]. In this model, next to the PMT factors, also evoked anxiety is a significant driver of activation. Also, the EPPM has been extensively applied in health communication research [41,42,43] and breast cancer screening [21]. Not only messages, but also personal characteristics, can lead to evoked threat, efficacy, and anxiety following the exposure to a threatening situation or issue, and, indirectly, to activation. In the current study, a model is tested in which a number of personal characteristics of the targeted women have an influence on the PMT factors threat and efficacy and on evoked anxiety which, in turn, have an effect on activation (Figure 1) [44]. 

Previous research only partially and indirectly documents the mediating effect of evoked threat, efficacy and anxiety on the relationship between personal characteristics and health behavior activation. Hong [45] found a negative association between health awareness and perceived susceptibility to a health threat. Nabi et al. [46] found that men high in perceived knowledge were more persuaded by an efficacy-only message for testicular self-examination, whereas those low in knowledge were not. Women high in perceived knowledge had comparable reactions to each of the different fear appeal messages for breast self-examination. It can be expected that individuals higher in AI will develop more anxiety and feelings of threat [11]. Neuberger et al. [47] found that self-efficacy, response efficacy, and mothers’ concern with breast cancer were significant predictors of intentions to engage in preventive behaviors with daughters. Kash et al. [34] report that higher anxiety was directly related to poor attendance at a clinical breast examination and poor adherence to monthly breast self-examination. These results suggest that a high risk perception may lead to less activation, and that this effect is mediated by developing high levels of anxiety. Because, in general, the mediating role of threat, efficacy, and breast cancer anxiety is unclear, the following research question is proposed: 

**Research question 1.** What is the mediating effect of perceived threat, perceived efficacy, and evoked anxiety on the relationship between the personal characteristics of women on the one hand, and breast cancer screening activation on the other?

## 3. Method

### 3.1. Procedure 

In May 2015, all 7000 women aged 50 to 69 (and thus eligible for the free-of-charge breast cancer screening organized by the Flemish government), living in four different Flemish (Belgian) towns received an invitation to the free-of-charge breast cancer screening organized by the government, and also received a questionnaire. The study is thus based on a cross-sectional survey. The relevant population of these four towns together was fairly typical of the Flemish population in that age group. These women were invited by the government agency with a standard invitation letter to which the questionnaire was added. In this questionnaire, the variables discussed in the previous section were measured (see next section for measurement details). Seven hundreds of them returned it fully completed. 

### 3.2. Measures

A number of measurement scales are based on previously validated measures, others were self-developed. In Table A1, the items for each of the measures and the source of the scales are shown. The four dependent variables, Future info seeking, Forward to friend, Talk to doctor, and Screening attendance, are constructs that consist of a concrete singular object and a concrete attribute, and were therefore measured by means of single-item scales [48]. All but one independent variables (Health awareness, Need for Cognition, Affect Intensity, Breast cancer knowledge, Topic involvement) and the three mediators (Perceived threat, Perceived efficacy, and evoked Breast cancer anxiety) were measured by means of multi-item 5-point scales. The internal consistency of these multi-item scales was tested by means of a Cronbach alpha analysis. The alphas are also reported in Table A1. An alpha > 0.7 indicates sufficient internal consistency. Since all multi-item scales were internally consistent, the scores on the items were averaged and this average score was used in subsequent analyses. Perceived increased breast cancer risk was measured on a 0/1 scale. 

The questionnaire was pre-tested in a sample of women (*n* = 10) belonging to the target group of the main study. All analyses reported hereafter are based on information collected in the survey, apart from the actual screening attendance. The latter information was provided by the government agency, for all of the women who completed the survey. 

### 3.3. Sample Characteristics

The sample contains both first-time invitees and women who had already participated in the screening program before. However, this information was not disclosed by the government agency. Of the total sample, 41% of which belong to age group 50 to 54, 22% to age group 55–59, 22% to age group 60–64, and 15% to age group 65–69. Forty-two percent were educated beyond high school level and 38.2% participated in the breast cancer screening. The average scores on the personal characteristics are as follows: health awareness (3.62), need for cognition (3.19), affect intensity (3.47), breast cancer knowledge (1.34), topic involvement (3.67), and 42.4% indicated the perception to have an increased breast cancer risk. The personal characteristics are not highly correlated. There are significantly positive correlations between health awareness and affect intensity (r = 0.190), breast cancer knowledge (r = 0.119), and topic involvement (r = 0.314), between the need for cognition and breast cancer knowledge (r = 0.159), between affect intensity and topic involvement (r = 0.150), and between breast cancer knowledge and topic involvement (r = 0.185).

### 3.4. Analyses

Behind the relation between a predictor (antecedent) and an outcome (criterion), there may be a mechanism that “explains” the effect. For instance, in the context of the current study, a personal characteristic (for instance health awareness) may have an effect on an activation variable (for instance talking to a doctor) because health awareness leads to a higher perceived threat which, in turn, leads to a stronger intention to visit a doctor. In other words, perceived threat “explains” the relation between health awareness and the intention to visit a doctor. In this example, “perceived threat” is a mediator. A mediator accounts for the relation between the predictor and the criterion. Mediators explain how a predictor influences a criterion. They clarify what would otherwise remain a black box in terms of why an antecedent predicts an outcome. To test the model in Figure 1, mediation analysis was carried out using Hayes’ PROCESS macro model 4 [49]. Hayes’ PROCESS macros have become the standard approach to test mediation processes. 

In the current study, the effects of each of the six personal variables (antecedents) on each of the four activation variables are tested. In each model, the mediating effect of threat, efficacy, and breast cancer anxiety on the relation between personal characteristics and activation variables is simultaneously investigated (Figure 1). This implies 24 different model estimations. In each model estimation, two types of tests are crucial. The first test evaluates the direction, strength, and significance of the predictor (personal characteristic) on the outcome variable. This is called the direct effect. The second test evaluates the direction, strength, and significance of the indirect effect of the predictor on the outcome, through a mediator. There is an indirect effect test for each mediator, thus three per analysis. Full mediation means that the mediators explain the full effect of a personal characteristic on an outcome variable and there is thus no direct effect of the personal characteristic on the outcome variable. Partial mediation means that, apart from the explanatory power of the mediators, there is also a significant main effect of the personal characteristic on the outcome variable.

## 4. Results

In Table 1, the results are given for each of the 24 models. Only significant effects are reported. All non-significant effects have *p* > 0.05 values. 

Participants with higher health awareness report a higher intention to seek information and to talk to a doctor. However, participants with higher health awareness participated less in the screening program. Hypothesis 1 is partly supported. Threat fully mediates the effect of health awareness on forwarding to a friend, and partially mediates the effect of health awareness on talking to the doctor. Efficacy fully mediates the effect of health awareness on forwarding to a friend. Breast cancer anxiety partly mediates the effect of health awareness on future information seeking and fully mediates the effect of health awareness on forwarding to a friend.

There is a direct negative effect of need for cognition (NFC) on forwarding to a friend. The higher the need for cognition, the lower the intention to forward the message to a friend. Hypothesis 2 is not supported. Neither threat nor efficacy mediates the effect of NFC on any of the four activation variables. Breast cancer anxiety fully mediates the effect of NFC on future information seeking, partially mediates the effect of NFC on forwarding to a friend, and fully mediates the effect of NFC on talking to the doctor. 

Higher affect intensity leads to a higher intention for information seeking, forwarding the message to a friend, and talking to a doctor. There is no direct effect of affect intensity on breast cancer screening. Hypothesis 3 is supported, except for screening attendance. Threat partially mediates the effect of affect intensity on forwarding to friend and talking to the doctor. Efficacy partially mediates the effect of affect intensity on forwarding to friend. Breast cancer anxiety partially mediates the effect of affect intensity on future information seeking, forwarding to friend, and talking to the doctor. 

The higher the *breast cancer knowledge*, the higher the intention to talk to the doctor. Hypothesis 4 is only partly supported. None of the mediators mediate the effect of breast cancer knowledge on any of the four activation variables. 

A higher topic involvement leads to a higher intention to search for more information, forward the message to a friend, and talking to a doctor. Hypothesis 5 is confirmed, except for screening attendance. Threat and efficacy partially mediate the effect of topic involvement on forwarding to friend. Breast cancer anxiety partially mediates the effect of topic involvement on future info seeking and forwarding to friend. 

The higher the perceived risk, the higher the intention to search for breast cancer information to forward the message to a friend and to talk to a doctor. However, contrary to expectations, the higher the perceived risk, the lower the participation in the screening program. Hypothesis 6 is confirmed, except for screening attendance. Threat partially mediates the effect of perceived risk on forwarding to friend. Breast cancer anxiety partially mediates the effect of perceived risk on future info seeking and forwarding to friend.

## 5. Discussion

In general, health awareness, affect intensity, topic involvement, and perceived risk have the most profound effect on activation, while the effect of need for cognition and breast cancer knowledge is more limited. Breast cancer anxiety, and to a lesser extent evoked threat, have a substantial mediation effect, especially on forwarding the message to a friend, while efficacy perceptions seem to be less important for activation. 

However, the effect of personal characteristics on actual participation in the population based breast screening program is very limited, and sometimes in the unexpected negative direction. Increased health awareness leads to less participation in the free of charge population based breast screening program. Previous research in the area of skin cancer prevention also demonstrated that a good knowledge of skin cancer among adolescents does not affect sun bathing habits or the intention to change these habits [50,51]. The main reason for this negative effect of health awareness on screening attendance may be that many health care conscious women already attend opportunistic screening from a relatively young age onwards. For the population based screening, the health authorities and health policy makers remind the eligible population of the possibility to participate in relevant screenings. In opportunistic screening, the individuals are engaged through their own initiative or after a medical recommendation outside of the population based screening program. Indeed, 80% of the non-participants in the free of charge population based screening indicated that they had already participated in opportunistic screening, especially those in the younger age groups. 

In the present study, a higher need for cognition leads to a lower intention to forward the message to a friend. The effect of need for cognition is not mediated by perceived threat or efficacy, but only by feelings of anxiety. These results do not support previous findings [8,30,38,43]. NFC has a negative effect on feelings of breast cancer anxiety, and a higher breast cancer anxiety leads to more activation. Consequently, the lower an individual’s NFC, the more activated she will be, through developing more anxiety. The latter results, to a certain extent, supports the general tenet of NFC research that people low in NFC usually process stimuli affectively and are mainly not so much persuaded by carefully scrutinizing message content, but rather by a more peripheral and often affective message processing [8].

Although a higher level of perceived risk leads to more information seeking, forwarding the message to a friend, and talking to a doctor, it also leads to lower participation in the population based screening. These results only partially confirm earlier findings. A higher perceived risk has indeed been associated with more information seeking [20,30], but also with higher screening behavior [24,32]. However, some other studies show no relation [22,23,33], or even a negative relation [32], between risk perception and screening behavior. Again, in the current study, women with a high level of perceived breast cancer risk often participate in alternative screening programs already before the age of 50, and may therefore be less inclined to participate in the government-organized screening program. 

As messages are more persuasive if they are consistent with personal characteristics of the message recipients and the way that they process information, these findings can provide guidance for health policy decision makers and health communication professionals, to fine-tune communication to women about breast cancer screening. Based on our results, awareness campaigns should not focus too much on the efficacy of breast cancer screening, but rather on the threat of the disease and, even more importantly, on the anxiety and fear that the disease evokes. This is even more important for women who are highly health aware and affect intense, and who are highly involved with the topic and have a high risk perception. 

However, focusing too much on anxiety and fear in awareness campaigns may, in the longer run, not be the best strategy. While having a normal/moderate level of concern for the risk of any disease is healthy, being overly anxious or perceiving one’s risk as higher than it really is, can be just as negative as a lack of concern/worry/anxiety and perceiving one’s risk as lower than it actually is. A woman who engages in mammography screening because of anxiety may only be doing so to alleviate higher than normal levels of anxiety, and may engage in screening more often than is recommended. Not only may high levels of anxiety create unhealthy emotions for women and possible over utilization of screening, but also it seems unethical to promote this as a method to encourage women to engage in screening. Indeed, in general, the excessive use of fear appeals could install an atmosphere of anxiety that makes people very unhappy, or could even lead to reactance whereby people effectively ignore the message. While keeping on focusing upon the threat and the anxiety woman should feel when reading a breast cancer awareness message, a broader and more ethical strategy could be to focus on developing the personal characteristics that are the prime antecedents of breast cancer screening activation. Health campaigns could therefore focus on building stronger health awareness, breast cancer involvement and perceived risk, and use emotional arguments to appeal to those with a higher affect intensity. Correctly informing women about their risk of developing breast cancer is crucially important for screening participation. Encouraging them to talk to a doctor is a necessary first step. In that sense, developing a correct risk perception could be a crucial prerequisite to further screening behavior. 

In the current study, only one message was tested. Messages can be differently framed, for instance, positive or negative, rational or emotional, and endorsed by different ‘authorities (e.g., doctors, ex-patients). These different message frames could lead to different message processing, and hence also different levels of activation for people differing in personal characteristics [52,53,54]. For instance, Kao showed that high NFC individuals prefer negatively framed messages, whereas low NFC individuals prefer positively framed messages [55]. Geuens and De Pelsmacker found that high affect intense individuals express more positive attitudes and higher levels of enjoyment for a positive emotional appeal [11]. Kao found that less knowledgeable people develop a higher perceived risk and hence try to absorb more message information) [55]. Future research could thus further investigate the interaction between message framing and personal characteristics. 

## 6. Conclusions

More health awareness, and a higher affect intensity, topic involvement and perceived risk, have the strongest effects on activation, while the effect of need for cognition and breast cancer knowledge is more limited. Breast cancer anxiety, and to a lesser extent evoked threat, substantially mediate the effect of personal characteristics on activation, especially on forwarding the message to a friend, while efficacy perceptions seem to be less important for activation. Messages are more persuasive if they are consistent with the personal characteristics and the message processing style of the message recipients. The findings can thus provide guidance for health policy decision makers and health communication professionals to fine-tune their communication. Awareness campaigns should focus on developing a reasonable level of threat and anxiety and appeal to affective responses, and at least as more importantly, should develop higher levels of health awareness, breast cancer involvement, and a realistic breast cancer risk perception. Although these factors may not always immediately lead to actual screening participation, they do activate women to seek more information, talk to a doctor, or spread the message amongst their friends.

## Figures and Tables

**Table 1:** Results.

Independent Variable	Outcome Variable	Direct Effect	Indirect Effect through Mediator		
			Threat	Efficacy	Anxiety
Health awareness	Seek information	0.128 (*p* = 0.005)			0.054 (*p* < 0.001)
	Forward to a friend		0.025 (*p* = 0.007)	0.064 (*p* = 0.003)	0.041 (*p* = 0.001)
	Talk to a doctor	0.280 (*p* < 0.001)	0.019 (*p* = 0.030)		
	Attend screening	−0.225 (*p* = 0.039)			
Need for cognition	Seek information				−0.065 (*p* = 0.001)
	Forward to a friend	−0.225 (*p* < 0.001)			−0.038 (*p* = 0.011)
	Talk to a doctor				−0.035 (*p* = 0.023)
	Attend screening				
Affect intensity	Seek information	0.206 (*p* = 0.003)			0.062 (*p* < 0.001)
	Forward to a friend	0.138 (*p* = 0.025)	0.027 (*p* = 0.024)	0.089 (*p* = 0.006)	0.038 (*p* = 0.007)
	Talk to a doctor	0.246 (*p* < 0.001)	0.025 (*p* = 0.046)		0.028 (*p* = 0.046)
	Attend screening				
Breast cancer knowledge	Seek information				
	Forward to a friend				
	Talk to a doctor	0.445 (*p* < 0.001)			
	Attend screening				
Topic involvement	Seek information	0.437 (*p* < 0.001)			0.041 (*p* = 0.001)
	Forward to a friend	0.284 (*p* < 0.001)	0.034 (*p* = 0.034)	0.128 (*p* < 0.001)	0.026 (*p* = 0.011)
	Talk to a doctor	0.484 (*p* < 0.001)			
	Attend screening				
Perceived risk	Seek information	0.257 (*p* = 0.002)			0.058 (*p* = 0.005)
	Forward to a friend	0.245 (*p* < 0.001)	0.057 (*p* = 0.006)		0.033 (*p* = 0.026)
	Talk to a doctor	0.386 (*p* < 0.001)			
	Attend screening	−0.708 (*p* = 0.001)			

Note: cells are coefficients indicate direction and strength of the effect, significance levels are in parentheses.

## Appendix A

**Table A1:** Measures and items.

Measure	Items	Scale	Cronbach Alpha	Source
Future info seeking	I am planning to seek more information about breast cancer in the near future	1 (totally disagree) to 5 (totally agree)	-	Self-developed
Forward to a friend	I would share the message with a friend who did not have breast cancer before	1 (totally disagree) to 5 (totally agree)	-	Self-developed
Talk to a doctor	Breast cancer is a topic about which I will I will talk to my doctor	1 (totally disagree) to 5 (totally agree)	-	Self-developed
Screening attendance	Attended free-of-charge population breast cancer screening	attended screening or not (0/1)	-	Self-developed
Health awareness	I think a lot about my health I frequently ask questions about my health I pay attention to changes in my health I am very engaged with my health	1 (not at all characteristic for me) to 5 (very characteristic for me)	0.89	Self-developed
Need for cognition	15 items	1 (totally disagree) to 5 (totally agree)	0.77	Pieters et al. [25]
Affect intensity	20 items	1 (totally disagree) to 5 (totally agree)	0.82	Geuens and De Pelsmacker [27]
Breast cancer knowledge	Indicate which best corresponds with the amount of knowledge you have about breast cancer	1 (very little knowledge) to 5 (a lot of knowledge)	-	Self-developed
Topic involvement	I have a strong interest in breast cancer information Breast cancer information is important for me Breast cancer information is relevant for me	1 (not at all) to 5 (completely)	.84	Self-developed
Perceived risk	Are you aware of an increased risk for breast cancer compared to the average female?	Yes/no	-	Self-developed
Perceived threat	I am convinced that breast cancer is a very serious disease I am convinced that breast cancer can have serious negative consequences I am convinced that breast cancer, diagnosed at a late stage, often leads to amputation or is not curable Chances that I will get breast cancer are substantial I run a high risk of getting breast cancer It is quite likely that—as for many other women—I will also get breast cancer	1 (totally disagree) to 5 (totally agree)	0.71	Rogers [37]
Perceived efficacy	Screening strongly contributes to early diagnosis of breast cancer Screening is the most effective means to diagnose breast cancer in an early stage Screening decreases the risk of severe or advanced breast cancer Through breast cancer screening, I can avoid to suffer from a severe form of breast cancer I have the possibility to attend breast cancer screening and (in this manner) avoid [to suffer from] breast cancer	1 (totally disagree) to 5 (totally agree)	0.85	Rogers [37]
Breast cancer anxiety	If I think about breast cancer, I feel… Nervous mentally confused depressed my heart beat faster not at ease very anxious	1 (not at all) to 5 (completely)	0.94	Self-developed
